# Linezolid-Induced Lactic Acidosis: A Case Report

**DOI:** 10.7759/cureus.76618

**Published:** 2024-12-30

**Authors:** Moiuz Chaudhri, Eric Costanzo, Nusha Fareen, Mayurkumar Patel

**Affiliations:** 1 Internal Medicine, Ocean University Medical Center, Hackensack Meridian Health, Brick, USA; 2 Pulmonary and Critical Care Medicine, Jersey Shore University Medical Center, Hackensack Meridian Health, Neptune, USA; 3 Nephrology, Jersey Shore University Medical Center, Hackensack Meridian Health, Neptune, USA

**Keywords:** lactic acidosis, lfts, linezolid, mitochondria, mrsa

## Abstract

Linezolid, an oxazolidinone antibiotic, is widely used to treat infections caused by gram-positive bacteria that are resistant to other antibiotics. It inhibits bacterial protein synthesis by targeting rRNA. Although generally safe when used for short durations in uncomplicated patients, prolonged use may lead to adverse effects. Here, we present the case of a 66-year-old female with a complex orthopedic history, who developed a prosthetic knee infection unresponsive to other antibiotics. Consequently, she was treated with an extended course of linezolid therapy, which resulted in the development of lactic acidosis despite normal liver and kidney function.

## Introduction

Linezolid is a synthetic antimicrobial belonging to the oxazolidinone class, exhibiting strong activity against a wide range of gram-positive pathogens, including methicillin-resistant *Staphylococcus aureus *(MRSA) and vancomycin-resistant enterococci (VRE). While lactic acidosis following prolonged linezolid use has been documented, studies indicate that its complications can escalate to multiorgan failure and even death, with treatment durations ranging from one to 100 days [1,2]. Although linezolid generally has a mild side effect profile, including diarrhea, nausea, and headache, severe adverse effects such as pancytopenia, serotonin syndrome, peripheral neuropathy, and lactic acidosis have also been reported [3,4]. This case highlights the importance of recognizing linezolid-induced lactic acidosis, even in patients with normal liver and kidney function.

## Case presentation

A 66-year-old Hispanic female with a history of hypertension presented to our facility in February 2022. In January 2021, she underwent an open reduction and internal fixation of the left knee. The procedure was complicated by issues with her prosthetic knee, which was subsequently removed in April 2021. Intraoperative cultures and wound cultures identified *Mycobacteroides abscessus* and *Candida albicans*, respectively. She was treated with amikacin, tigecycline, fluconazole, clarithromycin, and linezolid until August 2021.

In October 2021, the patient was readmitted for worsening left knee pain and drainage. The orthopedic team removed the antibiotic spacer and placed a new one. Intraoperative cultures again grew *M. abscessus*. She was started on oral linezolid, clarithromycin, and IV amikacin to manage the infection.

On February 2, 2022, the patient presented to the hospital after experiencing an episode of vomiting. She was fully vaccinated against COVID-19 and, upon admission, tested positive for COVID-19 without symptoms. Her vital signs were stable, with a blood pressure of 156/72 mmHg, a temperature of 98.7°F, a heart rate of 90 bpm, a respiratory rate of 18 bpm, and an oxygen saturation of 98% on room air. Laboratory tests revealed an elevated lactic acid level of 4.9 mmol/L.

A CT scan of the abdomen and pelvis with contrast and a chest X-ray showed no abnormalities. The patient had no symptoms suggestive of an active infection and was not septic. It is noteworthy that she had been on linezolid since October 2021, which was discontinued upon admission to our facility on February 2, 2022. Following discontinuation of linezolid and administration of IV fluids, her lactic acid level improved to 1.6 mmol/L by day 2 (Table 1).

**Table 1:** Laboratory values throughout the course of treatment ALT: alanine transaminase; AST: aspartate transaminase; BUN: blood urea nitrogen

Characteristics	On admission	Day 1	Day 2	Reference values
BUN (mg/dl)	7	5	5	5.0-25 mg/dl
Creatinine (mg/dl)	0.4	0.3	0.3	0.4-1.0 mg/dl
AST (u/l)	20	14	17	10-42 u/l
ALT (u/l)	19	15	16	10-60 u/l
Lactic acid (mmol/l)	4.9	5.1	1.6	0.5-2.0 mmol/l

## Discussion

Linezolid was approved by the U.S. FDA in 2000 for treating infections such as hospital-acquired pneumonia caused by methicillin-susceptible *S. aureus*, MRSA, and *Streptococcus pneumoniae*, as well as community-acquired pneumonia [5,6]. It is also indicated for complicated and uncomplicated skin-related infections and infections caused by VRE [5].

Linezolid functions as a bacterial protein synthesis inhibitor by interfering with the translation of bacterial mRNA into proteins [7]. Unlike other inhibitors, it acts specifically at the initiation phase of protein synthesis. Linezolid binds to the 23S portion of the 50S ribosomal subunit, preventing the formation of the initiation complex [7]. Its similarity to human mitochondrial ribosomes may contribute to mitochondrial toxicity, leading to inhibition of protein synthesis and accumulation of lactic acid [8].

One of linezolid’s key advantages is its 100% bioavailability due to rapid absorption following oral administration [9]. However, linezolid-induced lactic acidosis is multifactorial in origin. The literature indicates that prolonged use exceeding three weeks significantly increases the risk of lactic acidosis [10], potentially due to mitochondrial ribosome similarity. Additionally, hepatic and renal dysfunction are major contributing factors, with patients experiencing these conditions being at higher risk [10]. Studies suggest that an estimated glomerular filtration rate of less than 30 ml/min is a significant risk factor for lactic acidosis (OR: 7.4; 95% CI: 1.0-8.44; p = 0.02) [8,11]. Furthermore, genetic polymorphisms in mitochondrial 16S rRNA, specifically A2706G and G3010A, have been identified as contributors to increased susceptibility to lactic acidosis [12-15].

Our patient had been on linezolid therapy for over four months before presenting to our facility, thereby increasing her risk of developing lactic acidosis. However, she exhibited normal liver function tests and kidney function (Table 1), making this an unusual presentation.

## Conclusions

Linezolid-induced lactic acidosis has a multifactorial etiology. Most reported cases occur in patients treated for more than three weeks, often in the context of liver or renal dysfunction. Although our patient had no preexisting comorbidities, the prolonged duration of therapy was a significant factor contributing to the development of lactic acidosis. This highlights the importance of recognizing the risk of linezolid-induced lactic acidosis even in patients without hepatorenal dysfunction, particularly when extended treatment is required.
